# Post-Mortem Microbiology: Retrospective Analysis of Infections Caused by *Enterococcus* Strains

**DOI:** 10.3390/pathogens11020204

**Published:** 2022-02-03

**Authors:** Katarzyna Jermakow, Marta Rorat

**Affiliations:** 1 Department of Microbiology, Wroclaw Medical University, Chalubinskiego 4, 50-368 Wroclaw, Poland; katarzyna.jermakow@umw.edu.pl; 2 Department of Forensic Medicine, Wroclaw Medical University, Mikulicza-Radeckiego 4, 50-345 Wroclaw, Poland

**Keywords:** autopsy, bacterial infection, differential diagnosis

## Abstract

Post-mortem microbiological tests are one of the basic methods for diagnosing the etiology of infections in forensic pathology. One of the major groups of microorganisms abundant in various parts of the human body during life and after death is *Enterococcus* spp. Depending on the area of the body involved and the patient’s condition, enterococci can be considered to be a microbiome, transient flora or a pathogen responsible for infection. The data used for the analysis were 12 medico-legal autopsy and microbiological reports. *Enterococcus* spp. was isolated in 10 out of 12 cultures of blood samples collected post-mortem. The abdominal origin of enterococci in the blood was detected in 8 cases. The non-abdominal origin of enterococci in the blood was associated with a skin and soft tissue infection, purulent pneumonia and infective endocarditis. These results suggest that enterococci may be considered a cause of severe infections and with high likelihood. Microbiological cultures are a valuable source of information for helping to confirm the cause of infection. Interpretation of the results of post-mortem examinations must be carried out on the basis of data collected before and after death with the participation of specialists from various fields.

## 1. Introduction

Microbiological tests are one of the basic methods for diagnosing the etiology of infections not only in clinical practice, but also in forensic pathology. During an autopsy, their use should be considered in cases of infection history, where unexplained inflammation is detected and when traumatic and non-infectious causes of death have been excluded. Unfortunately, microbiological procedures are still underused when determining causes of death. In practice, such diagnostic procedures are underutilized, they are mostly performed in the event of sudden infant death syndrome (SIDS), unexplained hospital deaths involving possible medical malpractice (misdiagnoses or inadequate treatment) or presence of infectious symptoms before a subject’s death [1,2,3].

Microbiological cultures carried out for sick patients can provide a lot of useful data. However, they sometimes raise some doubts. The results obtained are not always easy to interpret for clinicians, mostly because one of the most common difficulties is distinguishing colonization from infection. Sometimes in the absence of symptoms of disease, bacteria are isolated, while on other occasions, in the case of acute symptoms of an infectious disease, microbial cultures are negative. It is even more difficult to interpret post-mortem microbiological tests [1,2,3,4,5,6], the quality of which depends on many factors, i.e., post-mortem interval, antimicrobial treatment and methods used during the collection of materials (specimens obtained in a non-sterile manner leading to the contamination of samples). The method of handling the body after death, the nursing procedures carried out, the presence of vascular and urinary catheters and the corpse storage conditions may also affect microbiological results. All the abovementioned activities can mechanically transfer microorganisms that constantly or periodically colonize one part of the body to other areas, reducing the quality of the results and making the final assessment difficult.

In order to maintain the highest-quality results and to make post-mortem microbiology a valuable tool in determining the cause of infection, samples should be collected as soon as possible after death under conditions similar to those applied to live subjects. In addition, the results of microbiological post-mortem examinations should always be interpreted in conjunction with the circumstances of death, diagnostic results collected during the patient’s lifetime (if any), macroscopic pathological findings obtained during autopsy and histopathological examinations [1,3,7,8,9,10]. In all of these cases, consultations between the forensic pathologist and an experienced microbiologist are extremely useful.

One of the major groups of microorganisms abundant in various parts of the human body during life and after death is *Enterococcus spp*. These bacteria are often isolated from clinical materials, and depending on the area of the body involved and the patient’s condition, they can be considered to be a microbiome (a physiological flora), transient flora (which periodically colonizes in the human body) or a pathogen responsible for the development of various diseases. Enterococci are mostly natural inhabitants of the intestinal tracts of humans. The most common species are *Enterococcus faecalis* and *Enterococcus faecium.* They are particularly responsible for urinary tract, intra-abdominal and wound infections (e.g., bedsores, burns, ulcerations or surgical wounds), all of which in turn may lead to bacteremia and sepsis. Enterococci are also important etiological agents of infective endocarditis (IE). The problem in the treatment of enterococcal infections is their sensitivity to a relatively limited group of antibiotics, the list of which is constantly shortening due to the acquisition of resistance to β-lactams, aminoglycosides (HLAR: high-level aminoglycoside resistance), glycopeptides (VRE: vancomycin resistant enterococci) and oxazolidinones (LRE: linezolid resistant enterococci) [11].

The frequency of enterococcal isolation in the case of nosocomial and community-acquired infections is increasing. This phenomenon is closely related to their resistance to many classes of antibiotics and selection during antibiotic therapies. After such antibiotic therapies, naturally resistant enterococci begin to dominate the intestinal flora for many weeks, becoming a source of subsequent infections [11]. In intra-abdominal infections, where enterococci may co-exist with other microorganisms, their pathogenic role has long been questioned. Currently, the isolation of enterococci from the patient’s blood is considered to be a probable sign of a severe abdominal infection (peritonitis or intra-abdominal abscesses) or bacterial infective endocarditis (enterococci are a significant cause accounting for about 10% of all cases of infective endocarditis) [11,12]. Just like in clinical medicine, a negative blood culture does not rule out an infection. In this case, a detailed analysis of the culture result is needed to determine whether it is the cause of infection or a consequence of the translocation.

The aim of the study was to assess the significance of species from the *Enterococcus* genus in post-mortem microbiology cultures and to analyze their impact on death in cases of suspected medical malpractice. Accurate determination of the etiological factor of infection and its source may be of key importance for the legal liability of doctors if there is a suspicion of inadequate diagnostics and treatment or nosocomial infection. The discovery of a small, localized, hard-to-detect infection, especially when caused by bacterial strains resistant to many groups of antibiotics or located in a tissue with poor penetration of the drug, is an important defense argument.

## 2. Results

**Figure 1:**
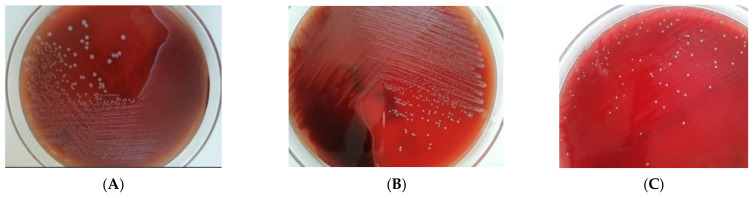
Different patterns of microbial growth in post-mortem microbiology culture. (**A**) Culture of cardiac blood sample from an infant (case #1) with abundant growth of *Enterococcus faecalis*. Death caused by a necrotic volvulus and secondary bacteremia. (**B**) Blood sample culture (case #3) with abundant growth of *Enterococcus gallinarum*. (**C**) Cerebrospinal fluid culture (case #3). False positive culture as a result of blood contamination during the collection procedure.

Among the decedents selected for more detailed examination, 11 out of 12 deaths occurred in the hospital; only one death, involving an infant (#1), occurred at home. In 10 blood sample cultures collected post-mortem, the presence of strains belonging to the genus *Enterococcus* was demonstrated (Figure 1 shows an example of a 24 h culture of blood and CSF—cerebrospinal fluid). In one patient (#2), accompanying microbial flora was also cultured. In the remaining nine cases, only one bacterial species was isolated from the blood samples. The dominant species was *Enterococcus faecalis*, which was isolated 5 times; on one occasion, the cause of infection was *Enterococcus gallinarum*.

Out of eight samples taken from CSF, five were convergent with the microorganism isolated from blood. In one case, when collecting a CSF sample by suboccipital puncture, the avoidance of blood aspiration was not possible (#3). In this case, the same microorganism as in the blood was isolated, although with a density much lower than that observed after sampling the blood (classified as blood contamination during CSF sampling; Figure 1B).

In 8 out of 12 cases, the following material was collected for culture: abdominal fluid/pus, abdominal swab, abdominal abscess swab, pancreatic abscess swab or spleen abscess swab. Enterococci were numerous (in terms of the number of colonies’ growth) and dominant in all abdominal specimens, but with mixed flora accompanying them (see Table 1). However, in the same cases, despite the isolation of mixed flora from abdominal materials, enterococci were isolated six times in the blood sample alone; the quantity was significant in all cases (the number of visible colonies on agar after 24 h incubation indicated a very high number of bacteria in the initial material, e.g., in the blood sample (see Figure 1A,B); in comparison, there was a small number of colonies from the CSF sample, and sparse growth (Figure 1C).

The non-abdominal origin of enterococci in the blood was observed in two cases: a purulent infection of skin and soft tissue after lower limb amputation (#2) and in a purulent pneumonia co-existing with myeloblastic syndrome (#4). In the case of a skin and soft tissue infection, *Klebsiella pneumoniae* ESBL+ was also isolated (both from the soft tissues’ smear and blood), and therefore, enterococci were considered to be responsible for a co-infection. In patient #4, the purulent infection in the lungs by the *Enterococcus. faecalis* etiology probably occurred as a result of chest trauma due to a traffic accident (enterococcal infection could have an exogenous origin).

CRP (C-reactive protein) was measured in 11 out of 12 cases, ranging from 0 to 100 mg/L. In patient #5, a high CRP concentration was detected on the day of death, and so, postmortem CRP testing was not undertaken. The lowest, but above the assumed range, CRP concentration was observed in the case of death caused by post-traumatic acute necrotizing pancreatitis complicated by spreading inflammation and necrosis on the abdominal cavity, without the presence of enterococci in the blood sample taken after death (#6; CRP 9.3 mg/L, post-mortem interval (PMI) 2 days). The highest CRP concentration was found post-mortem in patient #2 (CRP 100 mg/L, PMI 7 days) and was related to a mixed, purulent infection of the skin and soft tissue of the lower limb stump (etiological agents: *Enterococcus* and *Klebsiella*; both present in the blood collected post-mortem). The presence of *Enterococcus faecalis* in the blood from the heart, together with a high CRP concentration after death (66.3 mg/L, PMI 7 days), also occurred in the patient with multiple episodes of intestinal perforation (#7). In this case, spleen abscesses (a positive pus culture) and vegetations on the aortic valve and point-like lesions in the endocardium (indicating an infective endocarditis) were found during autopsy.

Post-mortem interval ranged from 2 to 13 days (mean 7 days). In all, 7 autopsies were performed within 7 days of death; 5 autopsies were performed more than 7 days after death (mean 10 days).

## 3. Discussion

In 9 out of 12 cases, only 1 bacterial species was isolated from the blood samples and elevated CRP concentration was observed. This situation improves the reliability of the culture results obtained and suggests that enterococci may be considered a cause of bacteremia. In most of the deaths analyzed, the source of enterococci in post-mortem blood samples was primary and secondary abdominal infections (peritonitis, intestinal obstruction, volvulus, intestinal necrosis, pyloric perforation, appendicitis and pancreatic or intra-abdominal abscesses). Therefore, it can be hypothesized that the source of these infections was enterococci representing the intestinal microbiome or periodically colonizing the patient’s gastrointestinal tract in hospital conditions (*Enterococcus* HLAR (high-level aminoglycoside resistance) or GRE (glycopeptide-resistant enterococci)). The involvement of enterococci in gastrointestinal infections is observed fairly frequent and well known to clinicians. Nevertheless, the pathogenic role in the development of these infections is more commonly attributed to Gram-negative intestinal bacteria (*Escherichia coli, Klebsiella, Enterobacter, Proteus*) and Gram-negative anaerobic bacteria (*Bacteroides fragilis* group). The deaths reported herein confirm that enterococci should be considered as an etiological agent in any case of severe intra-abdominal infection.

As shown in the literature, enterococci are a significant cause of infective endocarditis [12]. In the group analyzed, in one case, a post-mortem examination revealed the presence of vegetation on the valve (#7) and numerous bacteria of the *Enterococcus* genus. The presence of vegetation on the valve, found during forensic autopsy, should be considered a pathognomonic feature of the septic process developing in the host [9]. Case #7 was a man receiving psychiatric treatment who had a history of repeated self-harm behavior, manifesting in a tendency to swallow various objects, which in turn caused intestinal perforations and intra-abdominal abscesses (two spleen abscesses with a positive blood culture were detected post-mortem). It is highly probable that numerous episodes of intestinal perforation during the patient’s lifetime were the cause of the development of a cardiac vegetation (as observed after the death of the decedent). The post-mortem CRP concentration (66.3 mg/L) and the presence of the same species of enterococci in the blood of the decedent as those seen in the intra-abdominal abscess indicate an endogenous infection and potential impairment of the patient’s immune system mechanisms [5,12].

In one case (#4), bacteremia found after death was caused by severe purulent pneumonia. The involvement of *Enterococcus* *faecalis* as the cause of purulent pneumonia, confirmed by a positive pleural fluid culture, is extremely rare even in patients undergoing mechanical ventilation. One explanation could be the patient’s decreased immunity induced by myelodysplastic syndrome, which is associated with an increased risk of severe bacterial infections, including pneumonia. Furthermore, in immune-deficient persons, inflammatory reactions may be absent, and therefore, even bacteria with a low virulence potential may lead to severe infection and death [9]. In the abovementioned case, the infection was most likely exogenous (spread through the continuity of infected tissues) and developed following a chest trauma caused by a traffic accident. Much less likely is that pneumonia was caused by a different pathogen that was inhibited by antimicrobial therapy.

The case of the infant’s death at home (#1), associated with the presence of *Enterococcus*, was the most difficult to properly interpret. Despite the monoculture of numerous *Enterococcus faecalis* from a cardiac blood sample (Figure 1A), the post-mortem examination of a CRP concentration did not suggest any infection (CRP 0.0 mg/L) despite the autopsy results, which were conclusive. It seems unlikely that these bacteria would enter the bloodstream after death and multiply in a such large density under refrigerated conditions as 4 °C is the temperature inhibiting the growth/multiplication of most bacteria, including enterococci. The child died at home, a few hours before being transferred to the Forensic Department. The autopsy was performed four days after the death. Figure 1A shows the massive bacterial growth of *Enterococcus* *faecalis* after seeding two drops (approx. 100 µL) of blood taken from a few milliliters of pediatric cardiac blood. If the post-mortem spread of bacteria from the areas of their natural occurrence was present (post-mortem translocation), this would be expected to involve mixed intestinal flora and not a single bacterial species as in the abovementioned situation. This is in agreement with Christoffersen et al. [4] and Morris et al. [13], who indicate that post-mortem translocation usually involves mixed species of physiological flora. Pryce et al. [5] interpreted the results of post-mortem microbiological examinations obtained by specialists in the field of histopathology, microbiology, infectious-diseases, pediatric pathology and pediatrics. In most of them, cases of isolation of mixed bacteria or the presence of a culture-negative blood sample with the presence of the skin physiological flora in lung and/or spleen samples were considered to be post-mortem contamination. However, the growth of a single species of a common, strict pathogen in the blood and other materials collected from the infant after death was considered to be definitive confirmation of infection.

According to Palmiere et al. [10], there is no single, precise biomarker in post-mortem examinations that would definitely indicate the diagnosis of sepsis as the direct cause of death. CRP only has an auxiliary value, as its concentration depends on many factors of which the most important are post-mortem interval, hemolysis, autolysis, duration of treatment or immune system efficiency. Despite some disadvantages, CRP concentration seems to be a useful biochemical post-mortem marker, especially in sepsis [14,15,16]. Fujita et al. [6] observed the stability of post-mortem serum CRP concentration up to 48 h after death. They also determined that in traumatic death, survival time and the presence of severe infection were the major factors affecting the CRP increase. Regardless of the results of other diagnostics tests, for microbiologists, isolating one bacterial species in such a large density from blood indicates bacteremia. If, in addition, the species of isolated bacteria corresponds to that observed at a primary infection site (in case #1: intestinal necrosis), even a single isolation of such bacteria from the blood is considered to be a highly probable cause of bacteremia and/or sepsis in clinical conditions.

Considering all the abovementioned arguments and concepts, in the case we analyzed, we concluded that the presence of a massive growth of single enterococcal species in the infant’s cardiac blood was not accidental, did not result from post-mortem translocation and was not caused by contamination of the sample during incompetent (non-sterile) sampling for microbiological tests. It seems more likely that *Enterococcus faecalis* entered the infant’s blood due to extensive necrosis of the small intestine during their lifetime (leakage from the necrotic part of the intestinal wall), causing secondary bacteremia and indirectly causing the death of the infant. Why then was the CRP concentration, which traditionally indicates the presence of microbial infection, not elevated? One explanation for the lack of CRP may be related to the dynamics involved in the production of this protein—the concentration increases within 6 h after the onset of the inflammatory-inducing factor, reaching its peak after approx. 48 h [6]. The infant may have died before CRP increased to detectable and significant values. Not without significance is the fact that the case discussed concerns a two-month-old infant, in which the immature immune system may be responsible for the lack of an adequate inflammatory response, as already indicated by other authors [4]. Fujita et al. [6] performed post-mortem analysis of CRP concentration in acute and non-acute deaths. The lowest CRP concentration was observed in acute and immediate deaths (within 6 h; median value 0.1 mg/dL). Additionally, Ondrushka et al. [16] determined that CRP concentration does not exceed the norm (the post-mortem threshold level was set at 10 mg/L) in the early post-mortem period if it was not already exceeded at the time of death. In our study, the PMI varied, and the cut-off value was established at 5 mg/L, making comparison with other studies difficult, although in all cases—except #1—CRP was clearly elevated (9.2 to 100 mg/L). The concentrations of CRP determined after death were significantly lower than those obtained from live patients (93–300 mg/L), which we associate with the natural process of degradation of these proteins.

In one case (#2), more than one species of bacteria was detected in the post-mortem blood samples, and soft tissue inflammation of the stump was considered a factor initiating subsequent development of sepsis. In this patient, more species of bacteria were found in the blood than at the primary infection site. *Enterococcus* and *Klebsiella* were isolated from the purulent lesion of the stump, and in the blood, apart from the abovementioned bacteria, *Enterococcus* *gallinarum*, *Streptococcus uberis* and *Escherichia coli* were also observed (but at a much lower density). Many of these bacterial strains had acquired β-lactam and vancomycin resistance, suggesting prior colonization of the decedent in the hospital environment. The most likely cause of this mixed culture was (1) a purulent infection and (2) the insufficiently long, and therefore incorrect, procedure of disinfecting the skin of the decedent before collecting blood samples. Although Tang et al. [17] and Dermenigu et al. [9] indicate that a polymicrobial bloodstream infection is possible, which additionally increases the risk of death, this concerns infections produced by two bacterial species and not five as observed in our case. The mixed bloodstream infection can occur in long-term hospitalized patients, for example. We considered this case a mixed bloodstream infection, especially as a high CRP concentration (100 mg/L) was observed after death. The three bacterial species in the blood sample constituting a minority may indicate contamination of the sample during post-mortem collection. This contamination may most likely come from the environment [17], in this case from the skin of the decedent.

In the cases analyzed, antibiotics were used in most of the subjects, including five with a broad-spectrum antibiotic therapy administered immediately before death. Nevertheless, in four of them, the post-mortem blood sample was culture-positive, and the post-mortem CRP concentration was significant. Therefore, it can be concluded that, similar to the studies by Tang et al. [17], ante-mortem antibiotic therapy had no insignificant impact on the post-mortem microbiological culture results.

Case #3 is interesting as it highlights the possibility of endogenous enterococci being involved in the host’s pathological processes. Although endogenous, the isolated strain (*Enterococcus* *gallinarum*) was naturally resistant to vancomycin. Earlier antibiotic therapies, associated with numerous abdominal surgeries that the patient underwent, led to the selection of resistant strains inhabiting the intestines (naturally resistant to cephalosporins and vancomycin). The neoplastic process, carcinoid tumor of the appendix with liver metastases, facilitated the invasion of *Enterococcus* *gallinarum* from the intestines into the peritoneum and led to fatal sepsis.

Generally, resistant *Enterococcus* strains (HLAR, VRE, GRE) easily colonize a patient’s gastrointestinal tract during hospitalization. In our material, they were grown in three cases of patients hospitalized for at least seven days, including one in the intensive care unit; all were treated with antibiotics. An unquestionable example of nosocomial infection is case #11: death took place after 34 days in hospital, and strains of *Enterococcus faecium* GRE, typically from the hospital environment, were isolated from the post-mortem materials (blood, wound swab, abscess swab).

In each case of resistant strains isolated from post-mortem materials, colonization during hospitalization or nosocomial infection should be considered. Risk factors include long-term hospitalization, antibiotic therapy and admission to the intensive care unit. To confirm such a theory, the full medical documentation must be analyzed in detail. Bacterial resistance testing in post-mortem microbiology is not routinely performed, although it should be considered in cases of suspected nosocomial infection and medical error.

In our study, we only had two cases of negative blood cultures with a confirmed enterococcal infection. In the studies by Tang et al. [17], the susceptibility of bacteria to temperature declines and their inability to survive in a cold store were indicated as the main factor of negative blood cultures obtained post-mortem, although sensitivity to temperature fluctuations does not seem to be relevant to enterococci.

We also considered the PMI as a potential factor influencing the culture results. The small number of cases does not allow us to draw definitive conclusions. However, we did not observe more frequent occurrences of positive and mixed cultures with the longer PMI. Weber et al. [7] present similar results, although the study focused on cases of SUDI. Furthermore, the authors concluded that longer PMI may result in death of organisms and that PMI of several days is not associated with an increased risk of post-mortem translocation. In our material, in the two cases with the longest PMI (13 days), only one species of microorganism was cultured from the blood sample, which does not indicate a bacterial translocation. In two cases of negative blood cultures with concurrent positive polymicrobial culture of the abdominal fluid (# 6, # 9), the PMI was 2 and 10 days, respectively.

The results presented demonstrate the utility of post-mortem microbiological diagnostics in assessing the cause of the patient’s death as long as antiseptic rules are maintained. Only then is there an opportunity to distinguish an endogenous infection caused by physiological flora from an infection produced by nosocomial microbes. It is necessary not only to strictly adhere to the rules of collecting materials, selecting them correctly, using various methods in diagnostics to identify microorganisms and their sensitivity to antibiotics, but also to compare the results obtained with those received during a hospital stay. Additionally, it should be emphasized that all deaths selected for detailed analysis based on the post-mortem microbiological examinations require not only interpretation by an experienced microbiologist, but also the assessment of a forensic physician who takes all available data into account.

## 4. Materials and Methods

The source of data for the analysis was medico-legal autopsy and microbiological reports obtained by the Department of Forensic Medicine in Wroclaw Medical University issued in the years 2014–2021. Autopsies were carried out at the request of the prosecutor’s office in association with the suspicion of medical malpractice. During this period, 5797 forensic autopsies were performed, while material for microbiological culture was collected during 169 of them. Out of 41 cultures positive with *Enterococcus*, 12 were selected for further analysis. These were samples in which *Enterococcus* was present in monoculture in high numbers of colonies or as a clearly dominant species. The remaining 29 cases were discarded as examples of isolation of the natural human microbiome, e.g., *Enterococcus* in intestinal contents together with abundant gastrointestinal flora; temporary colonization of the skin, e.g., rectal skin swab, wound swab from an uninfected sacral decubitus wound; sparse growth of *Enterococcus* among other skin flora species; post-mortem translocation; and isolation of mixed microbial species including *Enterococcus* from naturally sterile materials such as blood, PMR, body cavity fluids, caused by post-mortem spread of bacteria from surrounding highly colonized sites.

**Figure 2:**
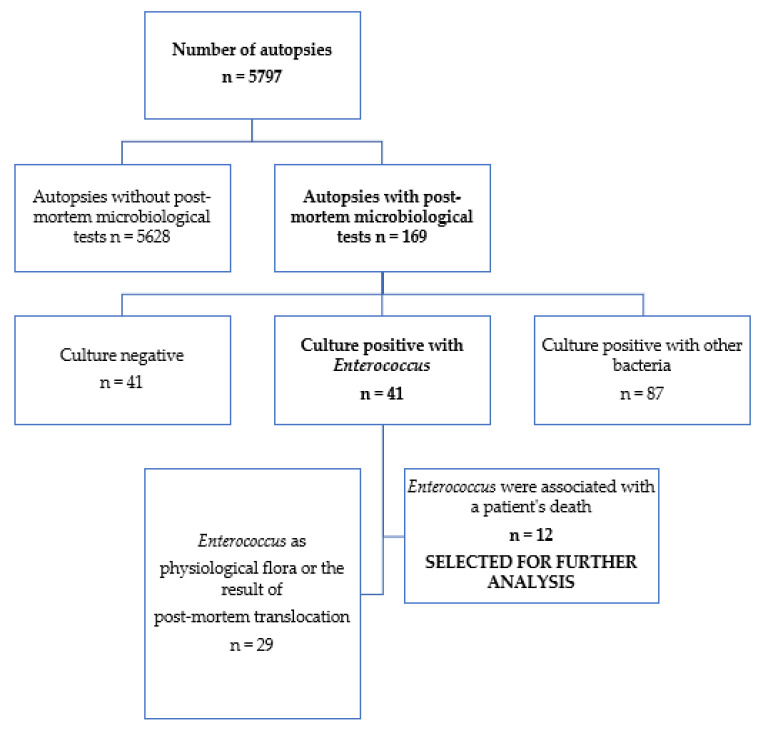
Flowchart of the cases with inclusion criteria.

After reviewing the results of the cultures performed, cases in which enterococci were associated with a patient’s death were selected for further detailed analysis (n = 12) (Figure 2). The results of microbiological tests were assessed in terms of species of microorganisms detected, the post-mortem macro- and microscopic examination as well as information from the medical records provided by the prosecutor (including the results of cultures and analytical tests).

Blood for post-mortem microbiological tests was collected in each case of suspected infectious cause of death, which was indicated by the data on the circumstances of death obtained from the authority conducting the proceedings. Other materials were collected at the pathologist’s discretion. Primarily sterile materials, such as blood and cerebrospinal fluid, were collected before opening the body, following the principles of asepsis and taking into account the need to extend the time of skin disinfection associated with the low temperature of the body. Blood samples were drawn with a sterile needle directly into a syringe, most often transthoracically from the cardiac chamber (8/12) or, in case of technical problems, from the femoral vessels (4/12) in accordance with applicable procedures. Immediately after the blood sample was taken, the needle was replaced with a new one (without needle cap removal to prevent contamination by microorganisms from the decedent’s skin and/or the environment). All CSF samples (*n* = 8) were collected via suboccipital puncture directly into the syringe. Similar to the blood sample, a needle exchange was used for transport. Other materials collected during selected autopsies for microbiological diagnostics were abdominal fluid/pus, abscess swab, pleural fluid, soft tissue swab/wound swab, abdominal cavity swab and bronchial swab. The decision to take additional microbiological samples was made by the forensic doctor during the autopsy. A detailed record of the cases is presented in Table 1.

The microbiological cultures of the samples obtained were performed within approximately 30 min (up to a maximum of 2 h) in the laboratory of the Department of Microbiology at Wroclaw Medical University. The post-mortem materials were seeded on microbiological media used classically in clinical diagnostics. The culture was performed for the detection of aerobic and microaerophilic bacteria using BD Columbia Agar with 5% Sheep Blood, BD MacConkey II Agar and BD Chocolate II Agar, while for anaerobic bacteria, the following media were used: BD Schaedler Agar with vitamin K1 and 5% Sheep Blood or BD Thioglycolate Medium with vitamin K and hemin. Culture media for detection of fungi included BD Sabouraud Agar with gentamicin and chloramphenicol and BD CHROMagar Candida. All the abovementioned media were obtained from Becton Dickinson (BD) (ready-to-use plated media). Bacterial growth was observed both after 24 h and 48 h of a 37 °C incubation, while anaerobic growth was observed for 7 days. For fungi, incubation was carried out for 72 h at 28 °C. Species were identified based on their biochemical characteristics. In all cases, commercial tests were carried out using classical biochemical methods (BD BBL Crystal Identification System, Becton Dickinson). In cases of difficulties with a precise determination of microbial species, identification of bacterial isolates was confirmed by matrix-assisted laser desorption ionization time-of-flight (MALDI-TOF; BRUKER). The sensitivity to antibiotics of selected bacterial strains was examined by a disc-diffusion test or E-test, according to EUCAST recommendations for a given calendar year (www.eucast.org, accessed on 5 November 2021).

The post-mortem examination of C-reactive protein concentration from the blood of the decedent was performed using an enzyme immunoassay (ELISA), using the commercial CRP EIA assay in accordance with the manufacturer’s instructions (DRG Instruments GmbH, Germany). The cut-off value for a positive result was established at 5 mg/L.

Limitations: The first limitation is the retrospective nature of the study; therefore, the authors had no influence on the method, type and number of collected samples. The second is the small number of analyzed cases, which is due to the rarity of enterococcal infections in forensic investigations (mainly medical malpractice cases). For this reason, the group is heterogeneous in terms of age, medical history, death circumstances and post-mortem interval. Because of the complexity of the cases, it is impossible to define categorically to what extent enterococci contributed to death. In addition, the sensitivity of CRP concentration in post-mortem diagnostics is limited due to many factors, such as hemolysis, autolysis and prior antibiotic therapy.

## 5. Conclusions

1. Enterococci can cause fatal infections and sepsis.
2. Microbiological cultures are a valuable source of information helping to confirm the cause of non-accidental deaths.
3. The credibility and usefulness of microbiological results depends significantly on sterile sampling of the appropriate material.
4. The source of sepsis should always be sought (in a similar manner as it is performed in a hospital setting). This knowledge is useful in assessing the correctness of medical management, thus determining the chance of a patient being cured.
5. Interpretation of the results of post-mortem examinations must be carried out on the basis of the largest possible amount of data before and after death (assessment of consistency with the current clinical picture) with the participation of specialists from various fields.

## Figures and Tables

**Table 1:** Baseline characteristics of cases studied; results of post-mortem microbiological tests and C-reactive protein concentration.

No.	Age, Years	Sex	History	Cause of Death	Sample for Microbiological Tests	Culture Results	CRP, mg/L	PMI, Days
#1	2 months	F	Death before admission to hospital	Small bowel volvulus and ischemia, necrosis of almost the entire small bowel, secondary bacteremia	Cardiac blood	***Enterococcus faecalis***	0.0	4
					Cerebrospinal fluid	***Enterococcus faecalis***		
#2	58	M	Hospital death (7th day)	Purulent skin and soft tissue infection of the lower limb stump complicated by sepsis and septic shock	Blood from the femoral vessels	***Enterococcus faecium VRE*** *Enterococcus gallinarum, Streptococcus uberis, Klebsiella pneumoniae ESBL, Escherichia coli ESBL*	100.0	7
					Soft tissues of the lower limb stump	***Enterococcus faecium VRE*** *Klebsiella pneumoniae ESBL*		
#3	68	M	Hospital death (6th day); surgery; ICU	Carcinoid tumor of the appendix with liver metastasis complicated by peritonitis and sepsis	Cardiac blood	***Enterococcus gallinarum***	12.0	3
					Cerebrospinal fluid (blood contamination)	***Enterococcus gallinarum***		
					Fluid from the pleural cavity	***Enterococcus gallinarum***		
					Abdominal swab	***Enterococcus gallinarum*** *Staphylococcus epidermidis*		
#4	79	M	Hospital death (23rd day); surgery; ICU	Craniocerebral trauma, chest trauma, massive purulent pneumonia	Cardiac blood	***Enterococcus faecalis HLAR***	57.9	8
					Cerebrospinal fluid	***Enterococcus faecalis HLAR*** *Staphylococcus epidermidis MR*		
					Fluid from the pleural cavity	***Enterococcus faecalis HLAR*** *Staphylococcus epidermidis MR*		
#5	63	M	Hospital death (34th day); surgery	Necrosis in the area of the gastrostomy, purulent bronchitis, peritonitis, sepsis	Blood from the femoral vessels	***Enterococcus faecalis***	Day of death 298.2	
					Cerebrospinal fluid	Negative		
					Abdominal pus	***Enterococcus faecalis*** *Klebsiella pneumoniae, Proteus mirabilis, Pseudomonas aeruginosa* *Candida albicans*		9
					Bronchial swab	*Klebsiella pneumoniae* *Pseudomonas aeruginosa*		
#6	7	F	Hospital death (18th day); surgery	Post-traumatic acute necrotizing pancreatitis complicated by expanding inflammation and necrosis on the abdominal cavity and sepsis	Cardiac blood	Negative	9.3	2
					Cerebrospinal fluid	Negative		
					Fluid from the pleural cavity	Negative		
					Abdominal fluid	***Enterococcus faecium*** *Stenotrophomonas maltophilia*		
#7	35	M	Hospital death (32nd day)	Pneumonia, splenic abscesses, myocarditis and infective endocarditis as a complication of multiple surgeries	Cardiac blood	***Enterococcus faecalis***	66.3	7
					Spleen abscess swab	***Enterococcus faecalis*** *Escherichia coli*		
#8	71	F	Hospital death (5th day); surgery; ICU	Acute intestinal ischemia and ileus complicated by sepsis	Blood from the femoral vessels	***Enterococcus faecium***	13.8	5
					Cerebrospinal fluid	***Enterococcus faecium***		
					Abdominal fluid	***Enterococcus faecium*** *MRSA,* *Candida albicans*		
					Urine	*Candida albicans*		
#9	53	F	Hospital death (2nd day)	Perforated peptic ulcer complicated by diffuse peritonitis	Blood from the femoral vessels	Negative	93.7	10
					Abdominal fluid	***Enterococcus faecalis*** *Streptococcus intermedius, Streptococcus vestibularis, Escherichia coli, Klebsiella pneumoniae, Enterobacter cloacae, Bacteroides fragilis, Bacteroides thetaiotaomicron, Clostridioides difficile*		
#10	56	M	Multiple hospitalizations before death	Massive hemorrhage from the area of hepatic hilum, purulent inflammation of the abdominal cavity after cholecystectomy	Cardiac blood	***Enterococcus faecalis***	33.7	
					Abdominal abscess swab	***Enterococcus faecalis*** *Escherichia coli, Klebsiella pneumoniae, Bacteroides fragilis,* *Candida albicans*		10
#11	57	F	Hospital death (34th day)	Multiple organ failure, pancreatic abscess	Cardiac blood	***Enterococcus faecium GRE***	65.0	13
					Cerebrospinal fluid	Negative		
					Post-operative wound swab	***Enterococcus faecium GRE*** *Hafnia alvei, Candida krusei*		
					Pancreatic abscess swab	***Enterococcus faecium GRE*** *Hafnia alvei, Leuconostoc sp.*		
#12	72	M	Hospital death (32nd day); ICU	Small cell lung carcinoma with liver metastasis complicated by acute liver failure and sepsis	Cardiac blood	***Enterococcus faecium***	13.0	2
					Cerebrospinal fluid	***Enterococcus faecium***		

Abbreviations: CRP—C-reactive protein, HLAR—high-level aminoglycoside resistance in enterococci, MRSA—methicillin-resistant *Staphylococcus aureus*, VRE—vancomycin-resistant enterococci, ESBL—extended spectrum beta-lactamases, GRE - glycopeptide-resistant enterococci, PMI—post-mortem interval; ICU—intensive care unit hospitalization; surgery—the patient underwent surgery during hospitalization prior to death; in the culture results, the dominant species are shown in bold.

## Data Availability

The data presented in this study are available from the first author on request.
